# Characterization of Partial and Near Full-Length Genomes of HIV-1 Strains Sampled from Recently Infected Individuals in São Paulo, Brazil

**DOI:** 10.1371/journal.pone.0025869

**Published:** 2011-10-14

**Authors:** Sabri Saeed Sanabani, Évelyn Regina de Souza Pastena, Antonio Charlys da Costa, Vanessa Pouza Martinez, Walter Kleine-Neto, Ana Carolina Soares de Oliveira, Mariana Melillo Sauer, Katia Cristina Bassichetto, Solange Maria Santos Oliveira, Helena Tomoko Iwashita Tomiyama, Ester Cerdeira Sabino, Esper Georges Kallas

**Affiliations:** 1 Division of Clinical Immunology and Allergy, Faculty of Medicine, University of São Paulo, São Paulo, Brazil; 2 Department of Translational Medicine, Federal University of São Paulo, São Paulo, Brazil; 3 Fundação Pro-Sangue, Blood Center of São Paulo, São Paulo, São Paulo, Brazil; 4 Department of Infectious Diseases, University of São Paulo, São Paulo, Brazil; 5 Public Health Department of São Paulo, São Paulo, Brazil; Johns Hopkins University Bloomberg School of Public Health, United States of America

## Abstract

**Background:**

Genetic variability is a major feature of human immunodeficiency virus type 1 (HIV-1) and is considered the key factor frustrating efforts to halt the HIV epidemic. A proper understanding of HIV-1 genomic diversity is a fundamental prerequisite for proper epidemiology, genetic diagnosis, and successful drugs and vaccines design. Here, we report on the partial and near full-length genomic (NFLG) variability of HIV-1 isolates from a well-characterized cohort of recently infected patients in São Paul, Brazil.

**Methodology:**

HIV-1 proviral DNA was extracted from the peripheral blood mononuclear cells of 113 participants. The NFLG and partial fragments were determined by overlapping nested PCR and direct sequencing. The data were phylogenetically analyzed.

**Results:**

Of the 113 samples (90.3% male; median age 31 years; 79.6% homosexual men) studied, 77 (68.1%) NFLGs and 32 (29.3%) partial fragments were successfully subtyped. Of the successfully subtyped sequences, 88 (80.7%) were subtype B sequences, 12 (11%) BF1 recombinants, 3 (2.8%) subtype C sequences, 2 (1.8%) BC recombinants and subclade F1 each, 1 (0.9%) CRF02 AG, and 1 (0.9%) CRF31 BC. Primary drug resistance mutations were observed in 14/101 (13.9%) of samples, with 5.9% being resistant to protease inhibitors and nucleoside reverse transcriptase inhibitors (NRTI) and 4.9% resistant to non-NRTIs. Predictions of viral tropism were determined for 86 individuals. X4 or X4 dual or mixed-tropic viruses (X4/DM) were seen in 26 (30.2%) of subjects. The proportion of X4 viruses in homosexuals was detected in 19/69 (27.5%).

**Conclusions:**

Our results confirm the existence of various HIV-1 subtypes circulating in São Paulo, and indicate that subtype B account for the majority of infections. Antiretroviral (ARV) drug resistance is relatively common among recently infected patients. The proportion of X4 viruses in homosexuals was significantly higher than the proportion seen in other study populations.

## Introduction

Human Immunodeficiency Virus (HIV) has several puzzling characteristics that separate it from other viruses. The immense genetic variability of HIV-1, which results mainly from the error-prone nature of its reverse transcriptase (3×10^−5^ mutations per nucleotide per replication cycle) [1]. The high rate of mutation and the rapid turnover of HIV-1 *in vivo* (10.3×10^9^ particles per day) [2] allows for the accumulation and fixation of a variety of host-immune response selected advantageous genetic changes in the virus population. These changes allow HIV-1 to resist the evolving defenses of the host. Recombination is another potential evolutionary source that significantly contributes to the genetic diversification of HIV and may produce more virulent viruses, drug resistant viruses, or viruses with altered cell tropism that may compromise the effectiveness of antiretroviral therapy and present major challenges for vaccine design [3].

The striking variability of HIV has led researchers to classify the virus into four phylogenetic distantly related groups: Main group (M), Outlier group (O), non-M-non-O group (N) and P group, which most likely reflect four independent events of cross-species transmission from chimpanzees [4], [5]. The M group, which dominates the current AIDS pandemic, is subdivided into subtypes (A–D, F–H, J and K), sub-subtypes (A1, A2, F1 and F2), circulating recombinant forms (CRFs) and unique recombinant forms (URFs) [4]. The molecular delineation of HIV-1 is a useful epidemiological instrument for tracking virus transmission and provides information about the patterns of genetic divergence that may have occurred during viral evolution.

HIV genetic variants are not geographically confined; however, there are circulating clades that are predominant in certain areas [6], [7]. For example, in Central Africa the main reported subtype is A and D, whereas other countries in Europe, USA, Australia and Thailand have reported subtype B as the main clade associated with their epidemic. Subtype C viruses are predominant in South Africa, Ethiopia and India, and CRF01_AE is the major circulating form in Southeast Asia [8], [9], [10]. The most prevalent HIV-1 subtypes in China, Japan's largest neighbor, are circulating BC recombinant forms, CRF07_BC, and CRF08_BC, and account for 50% of the HIV infected population, with subtype B HIV-1 accounting for 32% [11].

Brazil has the most populous nation in Latin America and the Caribbean and has the highest number of people living with HIV in the region (estimates vary from 460.000–810.000; UNAIDS/WHO. 2010 Report on the Global AIDS Epidemic; 2010). As in European countries and North America, HIV-1 subtype B is a major genetic clade circulating in Brazil, but the overall prevalence of non-B strains, particularly URF BF1, C and URF BC, has been increasing [12], [13], [14]. Data from recent studies of the near full-length genomes (NFLGs) of HIV-1 have provided evidence of the existence of Brazilian CRF strains designated as CRF28_BF, CRF29_BF, CRF39_BF, CRF40_BF, CRF46_BF and CRF31_BC (http://www.hiv.lanl.gov/content/sequence/HIV/CRFs/CRFs.html.)

Several studies have been conducted to develop and characterize panels of well-defined NFLG HIV-1 strains to be used as a resource in the evaluation of vaccine candidates [15], [16], [17]. In one of the largest studies conducted to date, Brown et al. [15] reported the complete genetic and biological characterization of a panel of 60 full-length sequenced HIV-1 isolates from 15 countries, including R5 and X4 viruses, representing clades A through D and CRF01_AE.

The present study involved the phylogenetic analysis of HIV-1 partial and NFLGs, the evaluation of HIV drug resistance and the evaluation of viral co-receptor tropism in treatment-naïve recently infected individuals from São Paulo, one of the main cities in Brazil.

## Materials and Methods

### Study population

One hundred and thirteen participants were selected from a larger cohort of 228 recently HIV-1 infected persons by order of entrance into the study. This sub-cohort was part of an ongoing project to investigate the host factors that contribute to immunodeficiency progression and has been previously described [18]. Briefly, recent HIV-1 infection was determined by the Serologic Testing Algorithm for Recent HIV Seroconversion (STARHS), and individuals were included in the study when they had a negative desensitized ELISA HIV-1 test, as that could indicate an incomplete antibody response as a consequence of recent HIV infection. For this study, patient PBMC samples were collected using standard procedures during their first clinical visit before the beginning of therapy and stored at −80°C until use. The patient data, including age, sexual orientation, number of CD4-positive T cells, and viral load were obtained from medical records. All study participants signed an informed consent form and the project was approved by the ethics committee of the federal University of São Paulo.

### Amplification and sequencing of HIV-1 DNA

The genomic DNA used for the PCR analyses was extracted from buffy coat samples with a QIAamp DNA Blood Mini Kit (Qiagen, Hilden, Germany), according to the manufacturer's instructions. Proviral DNA was used as the PCR template, as this allowed amplification of the NFLGs from five overlapping fragments as previously described [12], [13]. All amplification reactions were done in duplicate to eliminate PCR artifacts such as a sequenced NFLG being assembled from heterogeneous DNA targets. The amplified fragments were purified by use of a QIAquick PCR Purification Kit (Qiagen, Hilden, Germany) and directly sequenced on both strands using a variety of primer-directed strategies and the PRISM Big Dye Terminator Cycle Sequencing Ready Reaction Kit (Applied Biosystems/Perkin-Elmer, Foster City, CA) on an automated sequencer (ABI 3130, Applied Biosystems). After excluding the primer regions, the fragments for each amplicon were assembled into contiguous sequences and edited with the Sequencher program 4.7 (Gene Code Corp., Ann Arbor, MI).

### Screening for recombination events and identification of breakpoints

All sequences were screened for the presence of recombination events via the genotyping function of NCBI (NCBI Blast genotyping tool. Available at: http://www.ncbi.nih.gov/projects/genotyping/formpage.cgi) and the jumping profile Hidden Markov Model (jpHMM) [19]. Recombination events were further confirmed by the bootscanning method [20] implemented in SimPlot v. 3.5.1 for Windows [21] using the following parameters: window size 250 bp, step size 20 bp and the F84 model of evolution (Maximum likelihood) as a model to estimate nucleotide substitution, the transition\transversion ratio of 2.0, and a bootstrap of 100 trees. The significance threshold for the bootscan was set at 70%. The alignment of multiple sequences, including reference sequences representing subtypes A–D, F–H, J and K (http://hiv-web.lanl.gov), were performed using the CLUSTAL X program [22] and then followed by manual editing in the BioEdit Sequence Alignment Editor program [23]. Gaps and ambiguous positions were removed from the final alignment. The positions of crossover sites were preliminarily defined based on the distribution of informative sites, a method implemented in SimPlot v. 3.5.1, supporting the two incongruent topologies which maximize the χ^2^ value [24].

### Phylogenetic tree analysis

All phylogenetic trees were estimated by use of a general time reversible model under maximum likelihood (ML) using PHYML v.2.4.4 [25]. Heuristic tree searches using the ML optimality criterion were created using the nearest-neighbor interchange (NNI) branch-swapping algorithm. The approximate likelihood ratio test (aLRT) based on a Shimodaira-Hasegawa-like procedure was used as a statistical test to calculate branch support. All trees were displayed using either the MEGA v.5 package or the freely available Archaeopteryx Java software [26]. Nucleotide similarities were estimated using a maximum composite likelihood model implemented in MEGA version 5.0 software. The comparisons of tree topologies of the subtype B subgenomic regions were performed using an algorithm described by Nye et al. [27].

### Genotypic tropism analysis

For the predictions of HIV tropism, the *env* region identified in the NFLGs and partial-length *env* sequences that would encompass the V3 region were analyzed using a tropism prediction algorithm implemented as the web-based service geno2pheno [coreceptor] http://www.geno2pheno.org. To minimize the number of false predictions of CXC chemokine receptor 4 (CXCR4 or X4) tropic sequences as C–C chemokine receptor 5 (CCR5 or R5) tropic, a conservative false-positive rate (FPR) of 20% was used as a cutoff. Therefore, X4 or X4 dual/mixed-tropic viruses (X4/DM) were reported positive if their sequences had a prediction result FPR of≤20% or the 11/25 rule predicted a X4 virus, otherwise, they were considered R5-tropic viruses.

### Determination of drug-resistance mutations

The proviral nucleotide sequences of the *pol* region were translated into amino acid sequences and subsequently screened for drug-resistance mutations using the most updated WHO list for the surveillance of drug resistance mutations in antiretroviral-naive patients (SDRM version 2009, http://hivdb.stanford.edu/pages/WHOResistanceList.html).

All nucleotide sequences obtained during our study were reported to GenBank (Accession numbers pending).

## Results

### Samples

In total, 113 blood samples from recently HIV-1 infected individuals were subjected to NFLG sequencing. The median baseline HIV-1 viral load of 108 samples was 1.5×10^4^ copies/ml (range,<399−7×10^5^). The median baseline CD4 cell count was<560 cells/mm3 (range, 83–2449 cells/mm3) in 98.2% of individuals. The participants' ages ranged between 18 and 56 years, and the median age was 31 years. The majority of subjects were males (*n* = 102; 90.3%) and a sizable proportion (*n* = 90; 79.6%) reported transmission through homosexual contact. All patients were treatment-naive at the time of sample collection. The main characteristics of the study population are given in Table 1.

**Table 1:** Characteristics and main findings of the Brazilian HIV-1 recently infected individuals.

Male gender	102 (90.3%)
Media age (Years)	31
Risk group (*n* = 113)	
homosexual men	90 (79.6%)
heterosexuals	23 (20.4%)
Median CD4 count (cells/mm3)	560
Median viral load (log HIV RNA copies/mL) (*n* = 108)	15000
Drug resistance mutations (*n* = 101)	14 (13.9%)
PI	6 (5.9%)
NRTI	6 (5.9%)
NNRTI	5 (4.9%)
Subtype distributions (*n* = 109)	
B	88 (80.7%)
BF1	12 (11%)
C	3 (2.8%)
BC	2 (1.8%)
F1	2 (1.8%)
CRF31 BC	1 (0.9%)
CRF2 AG	1 (0.9%)
Prediction of viral tropism (n = 86)	
X4 viral tropism (pure or dual/mixed R5/X4)	26 (30.2%)

Virus variants with stop codons in different open reading frames were identified in five samples. A gross deletion of 433 (Nucleotide position from start of HXB2 genome; 5464–5896) and 487 bp (Nucleotide position from start of HXB2 genome; 1337–1823) were observed in isolate 04BR 1071 (characterized as subclade F1) and 04BR 1074 (characterized as subtype B), respectively. A sequence duplication of 322 bp (Nucleotide position from start of HXB2 genome; 8512–8826) was observed in isolate 03BR 2015 (characterized as subtype B). We are currently assessing the accuracy of the large deletions and insertions in the aforementioned RNA isolates via ultra-deep 454 pyrosequencing. None of these sequences, including those with stop codons, were omitted from the analysis.

### HIV variants and sequences

Sequences were obtained for all five overlapped fragments that cover the NFLGs of 77 participants. Partial sequences were obtained from at least one fragment derived from 32 samples as shown in Table 2. Only four samples did not amplify for any fragment. The amplification failure in these samples was likely not be due to subtype diversity, as our method is effective for the analysis of diverse HIV-1 strains.

**Table 2:** HIV-1 strain subtyping by partial sequences.

Sample ID	Sequence fragment					Subtype
	A _(546–2598)_	B1 _(2157–3791)_	B2 _(3236–5220)_	C _(4890–7808)_	D _(7719–9537)_	
03BR 1025	+	+	−	+	+	B
03BR 1032	−	−	−	+	−	B
03BR 1034	+	−	−	−	−	B
03BR 1037	+	−	+	−	−	C
03BR 1042	+	+	+	−	+	B
04BR 1050	+	+	−	+	+	B
04BR 1056	−	+	+	+	+	B
04BR 1058	−	+	−	−	−	B
04BR 1059	+	−	+	−	−	B
04BR 1062	+	+	−	+	−	BF1
04BR 1063	−	−	−	−	+	B
04BR 1069	+	+	−	−	+	B
05BR 1073	+	−	+	+	+	B
05BR 1086	+	+	−	−	−	B
05BR 1087	+	+	+	−	−	B
05BR 1088	−	+	+	+	+	B
05BR 1091	+	+	−	+	−	F1
05BR 1096	+	+	+	−	+	B
05BR 1099	−	+	−	−	+	B
05BR 1102	+	+	−	−	+	C
05BR 1105	+	+	+	−	+	B
05BR 1106	+	+	+	−	+	B
06BR 1113	+	+	+	−	+	B
06BR 1116	−	+	−	−	−	CRF31 BC
06BR 1118	−	+	+	−	+	BF1
06BR 1122	+	+	+	−	−	B
06BR 1123	+	+	−	+	+	B
03BR 2010	+	+	+	−	+	B
03BR 2013	−	+	+	−	−	B
04BR 2024	−	+	+	−	−	B
04BR 2030	+	+	+	−	−	B
05BR 2037	+	−	+	−	−	B

### The distribution of HIV-1 subtypes by NFLG

**Figure 1:**
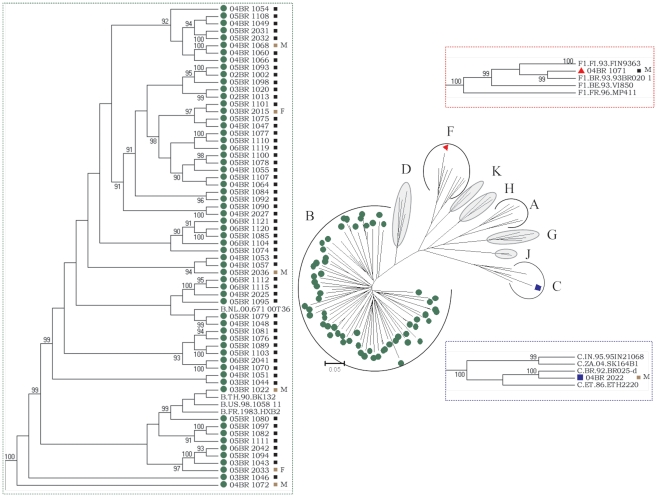
Phylogenetic tree constructed using a maximum-likelihood method from the NFLG sequences of 64 patient samples and 37 HIV-1 reference sequences from the Los Alamos HIV-1 database representing 11 genetic subtypes. Annotation of samples is as follows: symbol-green circle (subtype B), symbol-blue square (subtype C), symbol-red triangle (subclade F1), symbol-brown square-M indicates heterosexual male, symbol-brown square-F indicates heterosexual female, and symbol-black square indicates homosexual male. The approximate likelihood ratio test (aLRT) values of≥90% are indicated. The scale bar represents 0.05 nucleotide substitutions per site.

**Figure 2:**
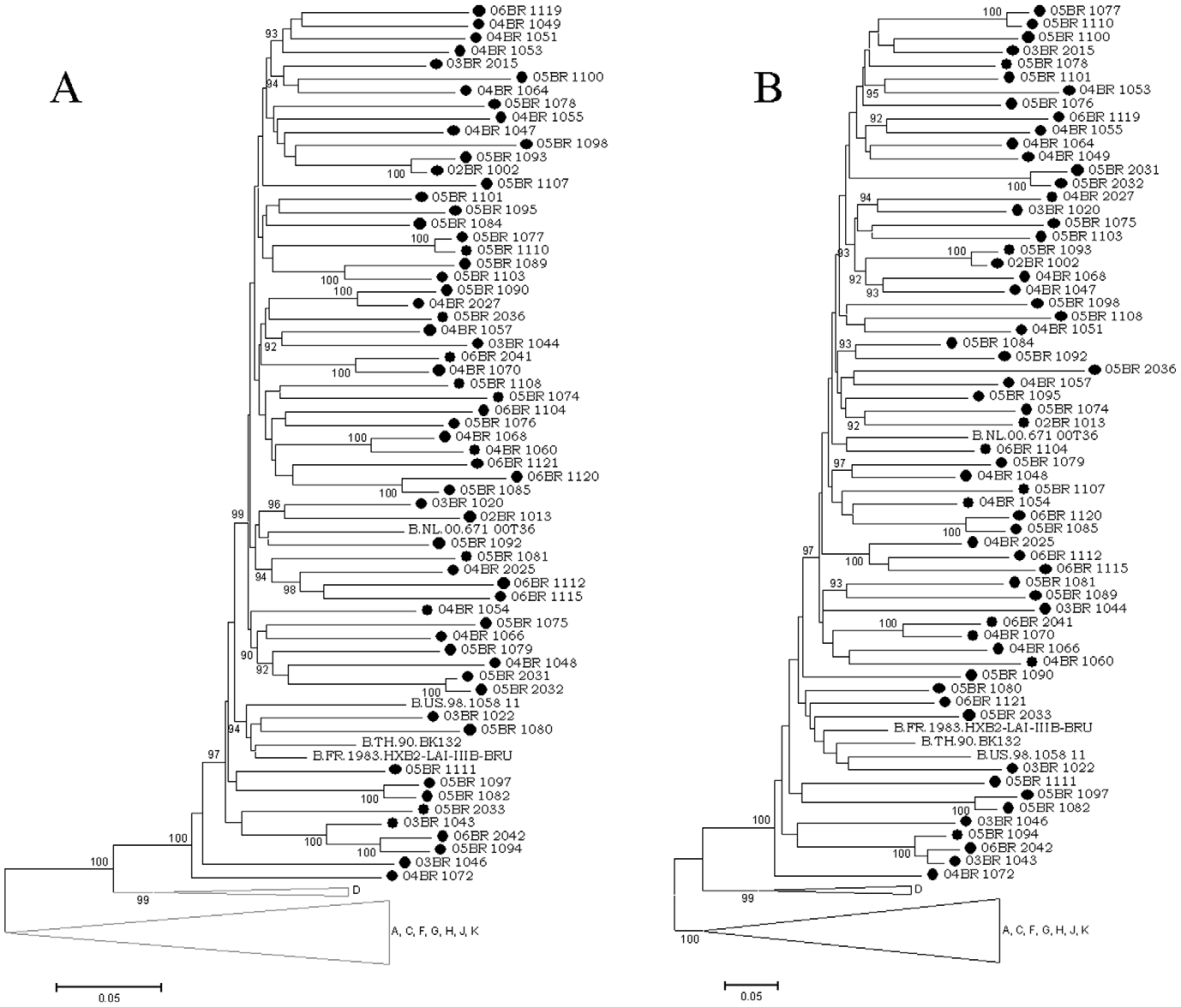
Maximum likelihood tree of sequences identified in this study (indicated by black circles) and reference strains inferred from full-length *gagpol* (A) and *env* (B) nucleotide sequences. For clarity purposes, the tree was midpoint rooted. The approximate likelihood ratio test (aLRT) values of≥90% are indicated at nodes. The scale bar represents 0.05 nucleotide substitutions per site.

**Figure 3:**
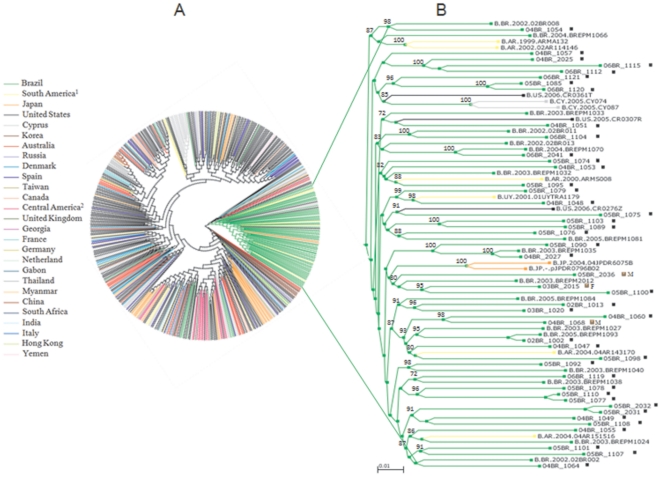
Phylogenetic relationships among subtype B HIV-1 NFLG sequences sampled from different countries. (a) Midpoint-rooted maximum-likelihood tree of 435 HIV-1 non recombinant subtype B NFLG sequences sampled from various global locations (colored branch). In addtion to previously published Brazilian subtype B NFLGs and other South American sequences from neighboring countries. The tree also contains 62 isolates sampled in São Paulo from HIV-1 recently infected patients over a 4- year period between 2002–2006 (the present datasets). (b) For clarity, the Brazilian isolates branched as a monophyletic cluster was displayed. Annotation of samples is as follows: symbol-brown square-F indicates heterosexual female, and symbol-black square indicates homosexual male. The approximate likelihood ratio test (aLRT) values of≥70% are indicated. The scale bar represents 0.01 nucleotide substitutions per site.

The phylogenetic analysis of the 77 HIV-1 incident samples with NFLG sequences revealed 64 pure subtypes (83.1%) and 13 recombinants subtypes (15.1%). A comparison of the NFLG sequences from 64 pure subtypes is presented in Figure 1. With the exception of isolates 04BR 2022 and 04BR 1071, which cluster with high aLRT values within the reference isolate clusters for subtype C and subclade F1, respectively, all samples clustered with subtype B. This group contains sequences from both MSM (*n* = 57) and heterosexual individuals (*n* = 7; 5 males and 2 females). In the topological phylogenetic tree of NFLG pure subtype B depicted in Figure 1; various samples are positioned in various well-defined clusters supported by high aLRT values. The stability and order of these clusters were further investigated with ML trees independently made with the *gag-pol* and *env* sequences using the same multiple genome alignment (Figures 2a&2b). The phylogenetic trees from both regions received an overall topological score of 63.8% as scored by the Nye et al. algorithm [27]. Close examination of both subgenomic trees revealed a shifting of topological position across numerous sequences in a manner suggestive of intra-subtype recombination [28]. For example, isolates 04BR 1060 and 04BR 1068 changed their topological positions over the *gag-pol* and *env* regions of their genomes. The computed topological score of the isolates in both regions was 33.3%, with a branch length mismatch of 89.5%. Similarly, isolate 05BR 1089 placed the *gag-pol* region with 05BR 1103 with an 100% of aLRT value, while *env* strongly grouped (aLRT<94%) with isolate 05BR 1081 (Figures 2a&2b). The evolutionary relationship between the representative subtype sequences was analyzed to illustrate the level of genetic diversity and to identify cross epidemic relationships that could suggest common geographic origins of subtype B present among presumably circulating subtype B viruses in São Paulo. The resulting 62 pure subtype B sequences were compared with each other and with 435 non-redundant B sequences (unique patient entries) available in the Los Alamos database and collected from more than 30 countries including Brazil (Figure 3a). The ML tree showed that sequences from Brazil appeared dispersed, even if a subcluster of Brazilian sequences was observed (Figure 3a; green nodes). This subcluster contains sequences from 73% (41/56) of MSM patient samples with non-recombinant clade B HIV-1 identified in this study. Further analysis of the subcluster indicated that some strains grouped with high aLRT (>98%) values with little genetic diversity (branch length<0.015) into various main transmission clusters composed of 2 to 3 infections per cluster within the B molecular subtype (Figure 3b). Notably, three sequences obtained from heterosexual contact-acquired infected patients (Figure 3b; brown square) are tightly positioned within some of the MSM risk groups. As shown in Figure 3A, the sequences identified in this study and other published Brazilian clade B sequences have a close genetic relationship with those previously found in the United States.

### Characterization of the NFLG recombinant sequences

**Figure 4:**
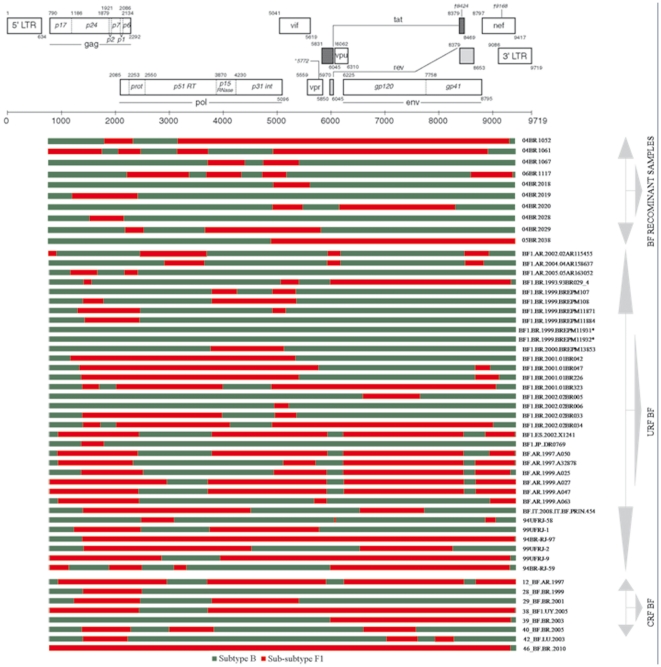
Schematic representation of the NFLG structure and breakpoint profiles of the BF1 sequences identified in this study and other BF1 URF and CRF published sequences. Sequences marked with the symbol (*) were originally classified as pure subtype but were characterized in the current analysis as pure B subtype, thus suggesting that a revised classification of these isolates in the GenBank and the HIV databases is appropriate. The region of subclade F1 and subtypes B are indicated at the bottom.

**Figure 5:**
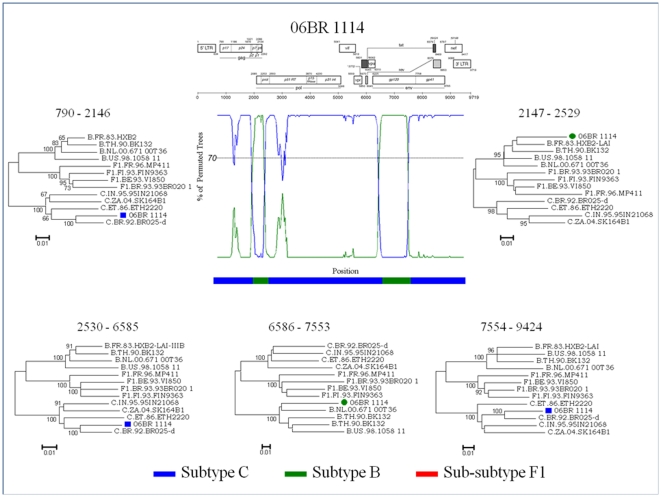
Exploratory tree analysis based on fragments between breakpoints as indicated by bootscan plot of isolate 06BR 1114. Annotation of samples is as follows: symbol-green circle (subtype B), symbol-blue square (subtype C). The approximate likelihood ratio test (aLRT) values of≥90% are indicated at nodes. The scale bar represents 0.01 nucleotide substitutions per site. The region of subtype C, subtypes B, and subclade F1 are indicated at the bottom.

Among the 13 NFLG isolates with strong evidence of recombination, 10 were mosaic isolates consisting of subtype BF1, 2 of subtype BC and 1 identified as CRF02 AG. None of the putative BF1 recombinants had a recombination profile identical to the other BF mosaics described in Brazil or elsewhere (Figure 4). Moreover, there was no obvious preferred location for breakpoints. To further test for recombination, ML phylogenetic trees were generated for the nucleotide sequence regions on either side of the breakpoints detected by the bootscan method (Figure 5). This analysis corroborates the results from the bootscan and thus provides unambiguous evidence for recombination events supported by high aLRT values. The nucleotide sequence of isolate 06BR 2039, characterized as CRF02 AG, was blasted against the NCBI nucleotide database. A significant number of similar full genome hit with a Nigerian HIV-1IbNg (GenBank: L39106) strain was indicated by the search.

### The distribution of HIV-1 subtypes by partial sequencing

The overall proportion of subtypes and recombinants generated by partial sequences is shown in Table 2. Consistent with the results obtained from the NFLGs analysis, the phylogenetic tree analysis of the sequenced fragments showed the predominance of subtypes B strains (79%). Subtypes C and BF1 were detected in four samples (6% each). Subtype F1 and a CRF31 BC variant were isolated in one sample (3% each).

### ARV drug resistance-associated mutations

The HIV-1 protease and RT regions of viruses obtained from 101 patients were scrutinized for any ARV drug resistance-associated mutations. Primary drug resistance mutations were detected in 14 patients (13.7%). Of these, 12 (85.7%) patients were infected with subtype B HIV-1. The distributions of these mutations by drug class were as follows: five patients had resistance to protease inhibitors (PIs), three patients to non-nucleoside RT inhibitors (NNRTI), three patients to nucleoside RT inhibitors (NRTI), one patient to PI+NNRTI, and two patients to NRTI+NNRTI (Table 3).

**Table 3:** Drug-resistance mutations detected in antiretroviral (ARV) naïve patients.

Sample ID	Resistance mutations			HIV-1 subtype	Tropism	Risk
	PI	NRTI	NNRTI			
04BR 1050	L24I,M46I,F53L, I54V, V82S	M41L, D67N, T215Y		B	X4	MSM
06BR 1123	D30N, M46I			B	R5	MSM
05BR 1105	D30N, M46I			B	ND	MSM
04BR 1055	D30N			B	R5	MSM
03BR 1020	G73S			B	R5	MSM
05BR 1077	V82A			B	R5	MSM
05BR 1088		V75A		B	X4	HET^M^ *
05BR 1075		M184V	K103N	B	R5	MSM
05BR 1107		M184I		B	R5	MSM
03BR 1042		M184I		B	ND	MSM
03BR 2018		M184V	K103N	BF1	R5	HET^M^
04BR 1053			K103N	B	X4	MSM
04BR 1067			K103N	BF1	R5	HET^M^
05BR 1111			K103N	B	X4	MSM

*HET ^M^; heterosexual man, MSM; men who have sex with men.

### V3 sequence analysis and viral tropism

An evaluation of the V3 loop amino acids and predictions of viral tropism were performed for 86 of the derived NFLG and partial sequences. Using the computer program geno2pheno [co receptor] for phenotype prediction, 26 patient virus strains (30.2%) were predicted to be X4 or X4/DM, and the V3 sequences of 60 patient virus strains (69.8%) were predicted to be R5-tropic virus. Among the MSM group evaluated for co-receptor tropism (*n* = 69), 19(27.5%) patient virus strains were predicted to be X4 or X4/DM and three patient samples have viruses with evidence of resistance to at least one ARV drug (Table 3). The tetrapeptide GWGR, a motif frequently detected in Brazilian subtype B sequences, in the V3 loop apex sequence was observed in 17 (19.8%) patients. Of these patients, 6 (25%) had a predicted X4 or X4/DM virus. The *N*-linked glycosylation sites (amino acids 4 to 6; positions 305 to 307 in HXB2 Env), essential for viral replication and the heptapeptide DIIGDIR (amino acids 32 to 38; positions 322 to 327 in HXB2 Env) were completely conserved in 64 and 19 subjects, respectively (data not shown).

## Discussion

In the present study, 77 NFLG and 32 partial sequences from recently HIV-1 infected Brazilian subjects were analyzed. The phylogenetic analysis of the sequences presented here showed that subtype B is largely dominant (79.8%), followed by BF1 URF. These results suggest the consistent and continual spread of subtype B and BF1 URF variants in São Paulo particularly among MSM, which are the most represented group in our study. Subtype C, F1, CRF31 BC and CRF02_AG were detected in small proportions. Overall, these data demonstrate a mature heterogeneous epidemic, which is consistent with the results of previous epidemiological studies in Brazil [29], [30], [31], [32], [33], [34]. In the phylogenetic analysis of NFLGs, there was evidence of various clusters of non-recombinant subtype B sequences. The majority of clustered subtype B isolates were closely related to the reference strains from United States. These results indicate that HIV infection in São Paulo is likely a result of direct transmission of a clade B virus from the United States, possibly via the heavy traffic of young Brazilian men in search of income opportunities in the United States.

The occurrence of intra-subtype recombination among non-recombinant subtype B isolates and the detection of the mixed BF1 and BC recombinants indicate that subtype B HIV-1 is not only the predominantly circulating subtype in the Brazilian HIV infected population, but the viral strains are also evolving and diverging into new strains over time. In addition, our analysis demonstrates that the heterogeneity within the global B subtype is equally reflected within the Brazilian subtype B viruses. Previous studies aimed at reconstructing the past population dynamics of subtype B in Brazil via the coalescent theory have indicated that the epidemic growth of this clade started in the late 1960 s, where it grew exponentially over the first 2 decades [35]. The element that initially contributed to and fueled the exponential growth of subtype B viruses may have been primarily be the transmission from acutely infected MSM and IDU [36], [37].

The proportion of recombinant strains in the state of São Paulo was defined from NFLGs and partial sequences. Sixteen recombinant variants have been found, in which two isolates 06BR 2039 and 06BR 1116 displayed a recombinant structure identical to CRF02 AG and CRF31 BC, respectively. These results indicate that at least 14% of the strains circulating in São Paulo were recombinants. Similar conclusions were previously highlighted in other studies in São Paulo [38].

In Brazil, on a regional scale, most infections in the frontier municipalities in the southern region of the country are caused by subtype C and BC recombinants, including CRF31 BC [12], [39], [40], which are also circulating as minor strains in the country's southeast and central west [32], [41]. Consistent with the results of these previous studies, our analysis also showed a lack of the substantial spread of subtype C and its recombinants in our cohort. It is possible that the high prevalence of subtype B viruses and other recombinant variants saturating the HIV-infected population coupled with effective behavior changes may be responsible for the differential spread of these strains. It is also possible that the bias created by the inclusion of predominantly MSM in our cohort affected the observed differences, and therefore, it may not reflect the HIV dynamics of the general population.

In agreement with the results of our previous study [42], the proportion of subclade F1 viruses examined in the present study was considerably lower than the previous estimates inferred from partial sequences. In fact, most HIV-1 subclade F1 strains circulating in Brazil may have contained recombinant genomes, particularly with subtype B as indicated in the present study and other studies [13], [43]. These recombinant genomes could have gone unrecognized by partial sequencing.

Our results indicate that prevalence of mutations conferring ARV resistance in recently infected patients is common (13.7%). It is possible that these resistant mutations may translate into lower rates of treatment response and higher rates of treatment failure. Some patients carrying multiple resistance mutations who fail ARV therapy have been shown to be infected with X4 viruses possessing a high replication capacity [44]. However, these observations could not be confirmed in other studies [45], [46]. The frequency of X4 and D/M tropism in the present study was 30.2%, a rate slightly higher than those found in a recent studies of HIV seroconverters [47] and drug-naive, chronically HIV-infected individuals [48], [49]. The high rate of X4 and D/M tropism seen in this study may be explained at least partly by the fact that most of the samples in this study were derived from MSM individuals. It is noteworthy that the prevalence of X4 and D/M tropism among our MSM group is high compared to the 3.2% reported in the study of 126 recently infected MSM from six major cities in the USA [50]. This high prevalence should seriously be considered when decisions are made about initial regimens for therapy-naive individuals, and HIV-1 coreceptor usage should be screened before initiation of any chemokine receptor CCR5 antagonists in clinical settings. These suggestions are in agreement with the conclusions of Frange et al. [51] that noted that X4/DM strains can heavily fuel the cellular HIV-1 reservoir leading to viral persistence over a long period complicating future therapeutic options, including CCR5 antagonists.

The assessment of HIV tropism in our study was limited to sequence- based algorithms rather than using phenotypic methods. Although phenotypic assays still have an edge over genotypic methods, genotypic predictors prove to be highly concordant with phenotype data and can reliably be used to determine viral tropism with better results in PBMC than in plasma samples [52]. In this study, we used geno2pheno because it allows for an adjustable cutoff, and it can determine HIV-1 co-receptor usage in all viral genotypes. This method has shown a similar performance to the Trofile phenotypic assay, the most often used tropism method [53]. Moreover, the method has been shown to achieve higher sensitivity while retaining high level of specificity when compared with the performance of different algorithms [54], [55].

Our study represents the largest analysis of the NFLG of the HIV-1 genomes undertaken to date from well-characterized recently infected patients and provides recent data on the molecular characterization of HIV-1 in treatment-naïve patients residing in São Paulo, Brazil. Overall, our results demonstrate a cocirculation of 3 group M viral subtypes (B, C, and F1), 3 URFs (BF1and BC), and 2 CRFs (CRF2 AG and CRF31 BC). However, these data need to be interpreted with some caution, as the samples recruited may not fully represent the general population of São Paulo because they disproportionately represent men who reported having sex with men.

The existence of various HIV-1 subtypes in Brazil will invariably challenge existing diagnostic tests and/or interpretation algorithms. Depending on future findings related to the transmissibility, pathogenicity, and treatment implications of various subtypes, these variant subtypes may also play a role in changing the nature of the HIV epidemic in Brazil. Thus, it is therefore to increase strain surveillance across Brazil. The identification and characterization of HIV-1 subtypes will strongly impact our understanding of the epidemic and the necessary approaches to ameliorate the epidemic. In conclusion, the present study addresses the need, in the design and implementation of vaccines and ARV therapy programs, for the identification of the concrete proportion of infections caused by different HIV-1 subtypes. Moreover, our data affirm that any new vaccines that will be pursued through clinical trials should take into consideration the multiple subtypes of HIV-1.
